# Out‐of‐pocket costs and burden among rural breast cancer survivors

**DOI:** 10.1002/cam4.1017

**Published:** 2017-02-23

**Authors:** Maria Pisu, Andres Azuero, Rachel Benz, Patrick McNees, Karen Meneses

**Affiliations:** ^1^ Division of Preventive MedicineSchool of MedicineUniversity of Alabama at BirminghamBirminghamAlabama; ^2^ Comprehensive Cancer CenterUniversity of Alabama at BirminghamBirminghamAlabama; ^3^ School of NursingUniversity of Alabama at BirminghamBirminghamAlabama; ^4^ School of Health ProfessionsUniversity of Alabama at BirminghamBirminghamAlabama; ^5^ Kirchner GroupBirminghamAlabama

**Keywords:** Burden, Cancer, costs, economics, out‐of‐pocket costs, rural, survivorship

## Abstract

Little is known about out‐of‐pocket (OOP) costs incurred for medical and health needs by rural breast cancer survivors and what factors may be associated with higher OOP costs and the associated economic burden. Data were examined for 432 survivors participating in the Rural Breast Cancer Survivor Intervention trial. OOP costs were collected using the Work and Finances Inventory survey at baseline and four assessments every 3 months. Mean and median OOP costs and burden (percent of monthly income spent on OOP costs) were reported and factors associated with OOP costs and burden identified with generalized linear models fitted with over‐dispersed gamma distributions and logarithmic links (OOP costs) and with beta distributions with logit link (OOP burden). OOP costs per month since the end of treatment were on average $232.7 (median $95.6), declined at the next assessment point to $186.5 (median $89.1), and thereafter remained at that level. Mean OOP burden was 9% at baseline and between 7% and 8% at the next assessments. Factors suggestive of contributing to higher OOP costs and OOP burden were the following: younger age, lower income, time in survivorship from diagnosis, and use of supportive services. OOP costs burden rural breast cancer survivors, particularly those who are younger and low income. Research should investigate the impact of OOP costs and interventions to reduce economic burden.

## Introduction

Patients with cancer, survivors, and their families incur considerable out‐of‐pocket costs for their medical and health needs regardless of healthcare insurance coverage 1, 2, 3, 4, 5. Cancer is associated with a higher risk of having medical care out‐of‐pocket (OOP) costs of $2000 or more per year, and of spending 20% or more of income on health care 1, 2, 4, 5, 6. In the 12 months after diagnosis, we found that breast cancer survivors reported paying OOP on average $316 per month in expenses for medical care, transportation, medical supplies, medications, and various needed services to improve or maintain quality of life 7. In the same population, Meneses et al. found that women reported on average three economic burden events including changes in income and economic lifestyle, borrowing money or using up savings, or in general sacrificing plans like vacations or other events 8. This burden is not without consequences and potentially affects immediate and future well‐being of survivors 8, 9, 10, 11. For example, the number of economic burden events was negatively associated with quality of life 8. Others have shown that survivors forego or delay medical care or medications because of cost, especially those who report cancer‐related financial problems 4, 12, 13.

The population of rural cancer survivors deserves some attention with regard to the OOP costs they may incur and the burden that these may cause. Rural cancer survivors report worse physical and mental health as well as being more likely to be unemployed due to health reasons than their urban counterparts 14, 15, 16. They also face several challenges in accessing care due to scarce medical resources and the need to travel longer distances to visit doctors and receive care 17, 18, 19. Rural residence and travel also affect the treatment choices patients with cancer make and the follow‐up care they receive post‐treatment 15, 17, 20. Moreover, poverty levels are higher in rural counties, and many rural residents are self‐employed or employed in small businesses facing challenges related to paying high insurance premiums and OOP expenses 17. Survivors residing in rural areas were more likely to forego health care because of cost, particularly those who were older than 65 years of age 21. In a survey of survivors in Ohio, about 25% of rural survivors reported needs related to paying medical bills and 21% needs related to health insurance compared to less than 18% and 13% of the urban survivors, respectively 22. Canadian rural cancer survivors were more likely to report travel costs and child care costs as important in treatment decisions compared to urban survivors 23. Currently, we do not know what OOP costs are incurred by rural cancer survivors for their long‐term well‐being, how burdensome these OOP costs may be, and what factors may lead to higher OOP costs and burden. Understanding more about these costs will allow for the design of interventions to manage such expenses and prevent rural cancer survivors from foregoing the care they need.

The objective of this study was to report the OOP costs of rural breast cancer survivors who participated in the Rural Breast Cancer Survivor Intervention (RBCS) trial 24, 25, 26. This trial tested the dissemination of an efficacious survivor‐centered, self‐management intervention addressing cancer treatment late effects, surveillance recommendations, healthy living after cancer, psychosocial concerns, and family, work, and insurance challenges among rural breast cancer survivors. Data from this trial allowed the unique opportunity to report on the OOP expenses of these rural survivors, an underserved population of which we know little about survivorship need and economic burden. We report OOP costs as well as OOP cost burden (i.e., the proportion of income spent on cancer‐related OOP costs). We further explore demographic, socioeconomic, and cancer characteristics that may be associated with OOP costs and OOP cost burden.

## Methods

This study was approved by Institutional Review Boards of the University of Alabama at Birmingham and the Florida Department of Health. Participants were women living in rural Florida diagnosed with Stage 0–III breast cancer, within the first three years after completing primary cancer treatment, at least 21 years of age, and with telephone or cell phone access. Rural eligibility was established based on residence in one of 33 Florida rural counties, or in a rural pocket of one of 34 Florida urban counties 27.

A total of 432 RBCS participants were randomized to either the Early Education and Support intervention group or the Delayed Education and Support intervention group. The RBCS four components included one intake assessment; three education and support sessions; one follow‐up education and support session; and six support calls. The early and delayed interventions had the same components, but participants in the delayed group received the support calls in the first 6 months after enrollment, and the education and support sessions at month 7.

### Measures

OOP costs incurred by survivors for cancer‐related medical care and other care to maintain or improve well‐being were collected using the Work and Finances Inventory (WFI) questionnaire. The WFI is a descriptive 46‐item survey, adapted and modified from Given et al. 28, with items asking about OOP costs incurred since the end of primary breast cancer treatment (baseline assessment), or since the last time the participant was surveyed (follow‐up assessments). Specifically, participants were asked about breast cancer‐related expenses that they, or their relative or any family member, spent OOP, excluding money repaid by insurance (whether they incurred any and, if so, how much in US dollars). The WFI also includes questions related to changes in family income and health insurance, work, and other economic events (e.g., filing for bankruptcy, unemployment, and loss of savings).

To assess participants’ characteristics and treatment, we used the Breast Cancer Survivor Socio‐demographic and Treatment Survey which includes questions on socio‐demographics (i.e., age, race, ethnicity, education level, marital status, type of health insurance, work status, and number of people living in the home) and questions on breast cancer treatment (i.e., months since diagnosis and months since end of treatment, type of breast cancer surgery, radiation therapy, chemotherapy, hormonal therapy, and/or anti‐HER2 therapy) 7, 8, 29. In addition, we measured depressive symptoms using the Centers for Epidemiologic Studies Depression Scale (CES‐D) 30. Scores ≥16 suggest clinically significant levels of psychological distress or depression 31.

Data were collected at baseline and at 3‐month intervals for a total of five assessment points over 12 months. OOP costs were grouped as follows:

1. *Medical care*, that is, expenses for hospital and doctor bills, emergency and usual care, medical supplies, prescriptions and over the counter drugs, physical therapy, and travel to the hospital, clinic, or doctor's office;
2. *Counseling and health maintenance*, that is, expenses for family or individual support and counseling, nutritional counseling, genetic testing and counseling, alternative treatments such as massage, herbs, alternative healers, and gym or health club memberships or other expense related to exercise;
3. *Side effect management,* that is, expenses for items such as wigs and prostheses;
4. *Home maintenance,* that is, expenses for help received for house cleaning, cooking. additional home maintenance such as yard work, and additional child care; and
5. *Other OOP costs*, that is, expenses related to insurance premium increases and other miscellaneous items.

## Analysis

We used a repeated measure model fitted with generalized linear mixed methods and a gamma distribution to explore which factors were associated with OOP costs across all time points 32. These included assessment time, demographics (i.e., age, marital status, race), socioeconomic status (i.e., education, employment, income, self‐reported income decrease since end of treatment, health insurance), and cancer factors (i.e., time since end of treatment, cancer treatment, use of prior or concurrent support services such as cancer support groups, counseling, or support websites). Predicted means were calculated for those factors significantly associated with OOP costs. The effect of the group assignment (Early vs. Delayed Education and Support) on OOP cost was examined as part of the main intervention evaluation. No relevant group assignment effect on OOP costs was found and, therefore, the combined sample was used in the analysis presented here.

At each assessment point, we computed OOP burden as the percent of monthly income spent on OOP cancer‐related costs. To explore which factors were associated with OOP burden, we used a repeated measure model fitted with generalized linear mixed methods and a beta distribution with logit link using the same predictors listed above. Predicted means were calculated for those factors significantly associated with OOP burden.

Six months after enrollment, 361 participants were retained (16% attrition). Due to an association between baseline self‐reported mental health and dropout risk 24, the impact of missing data on our analysis was mitigated by using linear mixed models and including an indicator for depression (baseline score for the CES‐D scale ≥16). Significance was held at the traditional 0.05 level. No correction for multiple testing was applied due to the exploratory rather than confirmatory nature of the analyses. All analyses were conducted using SAS v9.4 (SAS Institute Inc., 2013).

## Results

Almost half of the 432 rural breast cancer survivors in this study were 65 years old and older, and the majority was married or living with a partner and Caucasian (Table 1). About 25% had low education (high school or less), 45.4% were retired, and 34.8% had a family income of $40,000 or less, but most (94.4%) had health insurance, usually Medicare. About 80% were 12 months or more past the end of treatment with 23.4% being 25 months or more past that time point. The majority received surgery with radiation or surgery with chemotherapy and radiation and were on hormonal treatment.

**Table 1:** Baseline demographic, socioeconomic, and treatment characteristics of RBCS participants

Characteristic	*n* (%)
Age groups	
35–45	15 (3.5)
46–64	211 (48.8)
65–90	206 (47.7)
Marital status	
Never married	10 (2.3)
Married or living w/partner	316 (73.2)
Separated/divorced/widowed	106 (24.5)
Minority	25 (5.8)
Education	
<High school	24 (5.6)
High school grad.	97 (22.5)
Technical school/some college	149 (34.5)
Completed college	104 (24.1)
Postgraduate	58 (13.4)
Employment status	
Full‐time	111 (25.7)
Part‐time	62 (14.4)
Unemployed	17 (3.9)
Homemaker	24 (5.6)
Retired	196 (45.4)
Disability	22 (5.1)
Family income^a^	
$20,000 or less	71 (19.3)
$20,001 to $30,000	60 (16.3)
$30,001 to $40,000	34 (9.2)
$40,001 to $50,000	46 (12.5)
Greater than $50,000	157 (42.7)
Decrease in income since end of treatment	50 (11.6)
Health insurance	408 (94.4)
Months since end of treatment^b^	
≤12	88 (20.8)
13–24	236 (55.8)
25+	99 (23.4)
Treatment mix	
Surgery only	60 (13.9)
Surgery, chemotherapy	67 (15.5)
Surgery, radiation	122 (28.2)
Surgery, chemo, radiation	183 (42.4)
Hormonal therapy^c^	287 (66.4)
Depression symptoms (CESD≥16)	105 (24.3)
Support services^d^	98 (22.7)
Dropped out	100 (23.2)

a *n* = 64 women did not provide income information.

b *n* = 9 women did not provide end of treatment date.

c On hormonal treatment at the time the data were collected.

d Cancer support groups, counseling, support websites.

**Figure 1:**
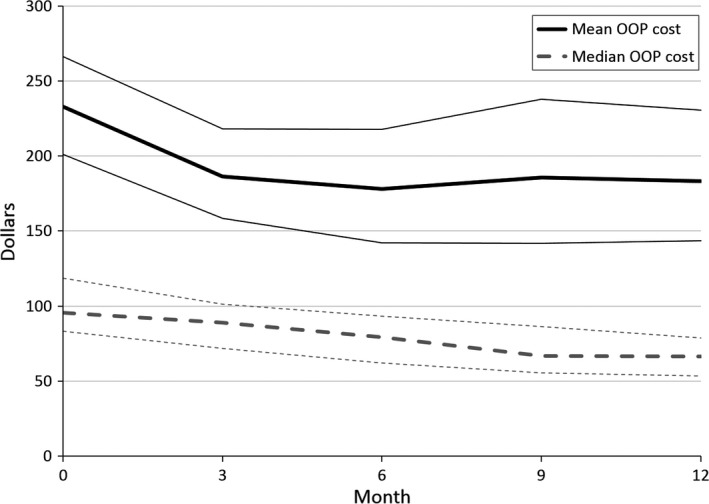
Mean, median, and bootstrap 95% confidence intervals for total monthly out‐of‐pocket (OOP) costs reported by study participants over the study period. Monthly cost in 2015 dollars.

OOP costs per month measured at baseline, representing monthly costs since the end of treatment, were on average $232.7 (median $95.6) (Fig. 1). The mean and median OOP costs declined from baseline to the next assessment point but remained constant after that (Fig. 1). At each assessment point, between 87.9% and 93.1% of participants reported costs for medical care, with means of $194.7 at baseline and between $129 to $147 in the rest of the assessments (Table 2). Between 18.6% and 27.7% reported OOP costs for counseling and health maintenance, with means of $78.1 at baseline and between $88.4 to $167.2 in the other assessments (Table 2). Between 2.1% and 23.0% reported OOP costs for side effect management, with means of $17.5 at baseline and between $38.5 to $58.6 in the follow‐up assessments (Table 2). Lastly, between 15.4% and 20.9% reported costs for home maintenance, with means of $111.7 at baseline and between $141.2 to $185.6 in the rest of the assessments (Table 2).

**Table 2:** Out‐of‐pocket (OOP) costs at 5 assessment points

Cost category	Self‐reported monthly OOP cost in 2015 dollars					
	*n* (%)	Mean	Median	Min.	Max.	SD
Baseline (*n* = 422)						
Medical care	393 (93.1)	194.7	82.7	0.2	1735.5	287.5
Counseling and health maintenance	117 (27.7)	78.1	30.9	0.2	1000.9	143.2
Side effect management	97 (23)	17.5	12.1	0.1	122.2	19.8
Home maintenance	88 (20.9)	111.7	79.2	0.5	833.3	128.6
Month 3 (*n* = 364)						
Medical care	326 (89.6)	149.3	71.1	0.3	1963.7	239.9
Counseling and health maintenance	76 (20.9)	88.4	39.1	0.1	703	126.5
Side effect management	23 (6.3)	38.5	36.9	0.1	122.3	34.3
Home maintenance	62 (17)	141.2	110.7	4.9	1576.8	213.6
Month 6 (*n* = 333)						
Medical care	297 (89.2)	129.3	57.5	0.1	2540.6	244
Counseling and health maintenance	67 (20.1)	146.9	41.5	0.1	3039.3	416.1
Side effect management	18 (5.4)	58.6	45.8	4.7	165.9	45.7
Home maintenance	53 (15.9)	137.6	91.5	9.6	591.3	121
Month 9 (*n* = 264)						
Medical care	232 (87.9)	133.2	50.4	0.5	1779.8	244.8
Counseling and health maintenance	49 (18.6)	167.2	43.2	0.1	4376.8	625.8
Side effect management	11 (4.2)	52.3	36.6	0.1	130	40.3
Home maintenance	41 (15.5)	185.6	111.4	7.4	1861.7	297.8
Month 12 (*n* = 331)						
Medical care	292 (88.2)	147.4	48.5	0.7	3528.5	337.8
Counseling and health maintenance	72 (21.8)	91.3	39.1	0.1	1079.8	166.4
Side effect management	7 (2.1)	55.1	16.7	5.6	195.5	72.2
Home maintenance	51 (15.4)	168.0	83.6	7.4	1782.4	271.6

**Figure 2:**
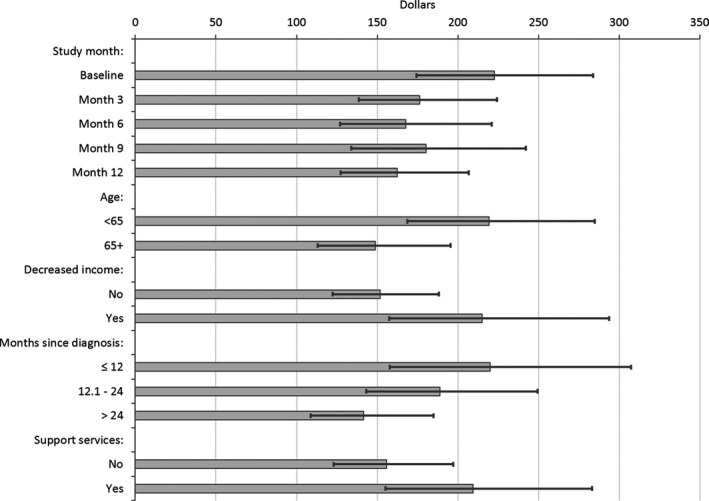
Predicted out‐of‐pocket (OOP) costs in 2015 US dollars (least squares means). Error bars represent 95% confidence intervals. Support services: cancer support groups, counseling, or support websites. Decreased income indicator reported at each time point.

The predicted mean OOP monthly costs were significantly higher at baseline ($222.5) than at the other assessment points (Fig. 2). Compared to Month 12, the mean ratio for OOP costs at baseline was 1.37 (CI: 1.13–1.66, Table 3). Across all assessments, participants who were younger than 65, had income decreases since diagnosis, were within 1 year of diagnosis, and used other supportive services had higher OOP costs (Fig. 2 and Table 3). The lowest predicted mean OOP cost was for participants who were more than 24 months from diagnosis ($141.7) and the highest was for OOP costs at baseline ($222.5), for survivors who were less than 12 months from diagnosis ($220.1) or younger than 65 ($219.3) (Fig. 2).

**Table 3:** Results of repeated measures model fitted to monthly out‐of‐pocket (OOP) costs and OOP cost burden

Characteristic	OOP costs	*P*	OOP cost burden	*P*
	Mean ratio (95% CI)^a^		Odds ratio (95% CI)^b^	
Assessment points				
Baseline	1.37 (1.13–1.66)	0.0211	1.38 (1.11–1.71)	0.007
Month 3	1.09 (0.93–1.27)		1.1 (0.89–1.35)	
Month 6	1.03 (0.88–1.22)		0.94 (0.78–1.13)	
Month 9	1.11 (0.92–1.34)		1.06 (0.84–1.36)	
Month 12	–		–	
Socioeconomic status				
Age ≥ 65	0.68 (0.52–0.88)	0.0038	0.68 (0.5–0.92)	0.0119
Married or partnered	0.82 (0.57–1.17)	0.2733	0.82 (0.57–1.2)	0.3094
Minority	1.11 (0.66–1.9)	0.6881	1.3 (0.7–2.41)	0.4024
Education				
<High school	0.53 (0.29–0.99)	0.2937	0.95 (0.44–2.03)	0.9722
High school grad.	0.83 (0.54–1.3)		1.05 (0.66–1.66)	
Technical/some college	0.76 (0.53–1.1)		0.93 (0.61–1.43)	
Completed college	0.78 (0.54–1.11)		0.95 (0.64–1.41)	
Postgraduate	–		–	
Employment				
Full‐time	–	0.0704	–	0.036
Part‐time	0.74 (0.49–1.12)		0.73 (0.45–1.18)	
Unemployed	0.57 (0.34–0.94)		0.55 (0.26–1.16)	
Homemaker	1.33 (0.78–2.24)		1.1 (0.59–2.05)	
Retired	0.92 (0.67–1.26)		0.72 (0.49–1.05)	
Disability	0.67 (0.41–1.11)		0.46 (0.28–0.74)	
Income				
$20,000 or less	0.69 (0.44–1.07)	0.3348	2.37 (1.5–3.73)	0.0012
$20,001 to $30,000	0.92 (0.62–1.38)		1.94 (1.24–3.03)	
$30,001 to $40,000	0.74 (0.51–1.08)		1.03 (0.68–1.57)	
$40,001 to $50,000	0.79 (0.53–1.16)		0.99 (0.66–1.49)	
Greater than $50,000	–		–	
Declined to answer	0.98 (0.66–1.45)		N/A	
Decrease in income since end of treatment^c^	1.42 (1.11–1.81)	0.0052	1.66 (1.21–2.29)	0.002
With health insurance	1.65 (0.99–2.75)	0.0552	1.7 (0.95–3.03)	0.0735
Clinical Status				
Months since treatment end				
≤12	1.55 (1.1–2.19)	0.043	1.47 (1.01–2.14)	0.1084
12.1–24	1.33 (0.98–1.82)		1.15 (0.82–1.61)	
>24	–		–	
Treatment mix				
Surgery only	0.88 (0.58–1.34)	0.1432	1.17 (0.71–1.95)	0.2998
Surgery, chemotherapy	0.9 (0.64–1.25)		0.92 (0.65–1.3)	
Surgery, radiation	0.7 (0.52–0.95)		0.8 (0.59–1.09)	
Surgery, chemo, radiation	–		–	
Using support services^c^	1.35 (1.03–1.76)	0.0318	1.46 (1.05–2.03)	0.0259
Depression CESD ≥16	1.05 (0.8–1.38)	0.738	1 (0.74–1.36)	0.9895

a Generalized linear mixed model for the natural log of the mean reported OOP cost.

b Generalized linear mixed model for the logit of the OOP cost as a proportion of income.

Decrease in income reported at each time point.

c Cancer support groups, counseling, support websites

**Figure 3:**
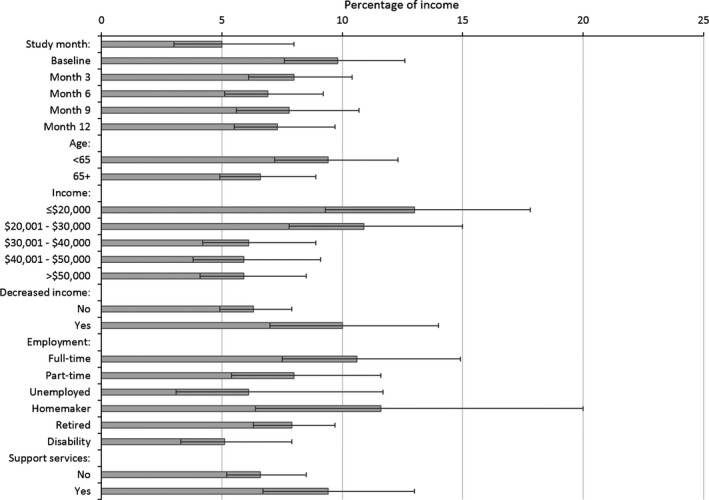
Predicted out‐of‐pocket (OOP) burden, that is, OOP cost as a proportion of income (least squares means). Error bars represent 95% confidence intervals. Support services: cancer support groups, counseling, or support websites. Decreased income indicator reported at each time point.

The predicted mean OOP burden (i.e., OOP costs as a percentage of income) was higher at baseline than in the other assessments (*P* = 0.007 Table 3). The predicted mean OOP burden was 9.8% at baseline, and between 7% and 8% in the other assessment months (Fig. 3). Across all assessments, younger participants, those with lower income, reporting income decreases since diagnosis, or using support services, had higher OOP burden (Table 3). Survivors with disability had lower burden than working survivors (Table 3). The lowest predicted OOP burden was for women on disability (5.1%), and the highest was for participants with incomes below $20,000 (13.0%) (Fig. 3).

## Discussion

Out‐of‐pocket costs after completing primary cancer treatment remained a substantial portion of the income of rural breast cancer survivors. Most of these costs were for medical care, including for hospital bills and medications, and declined over time as women progressed from diagnosis and into survivorship. However, these expenses are large and represent almost 13% of income for women with incomes below $20,000. These findings lead to a strong rationale for developing and testing interventions to address financial burden.

Cancer affects quality and quantity of life, and also the economic well‐being of survivors 5, 33, 34, 35, 36, 37, 38. The recent literature refers to patients’ costs as another side effect of cancer treatment, namely its “financial toxicity.”^39^, ^40^, ^41^, ^42^, ^43^ The Institute of Medicine identifies, as a tenet of high‐quality cancer care, the delivery to patients and families of “understandable information” including information on costs of cancer care 44. Similarly, we believe survivors should be aware of the costs to be incurred in survivorship. At a minimum, we should understand whether these costs act as barriers to obtaining recommended and needed care.

Our results are consistent with previous work showing that the financial toxicity of cancer does not end once primary treatment is over. Breast cancer survivors from Florida urban areas who had finished treatment and were within 2 years of diagnosis spent on average more than $300 per month in the period after diagnosis 7. This represented up to 30% of income for women with incomes below $20,000 7. Among the Florida rural breast cancer survivors analyzed here, survivors spent OOP for cancer‐related expenses on average more than $200 per month after treatment completion or up to about 13% of income for women with incomes below $20,000. The OOP costs reported in these two studies are somewhat different despite survivors being from the same state and responding to the same survey tool. However, in the first study, women were closer to diagnosis, and this may in part explain the difference. The lower costs of rural survivors may also be due to differences in healthcare‐seeking behavior. Given the longer distances to treatment centers, rural survivors may choose to obtain less care and, as a result, incur lower costs 17, 18, 19. Moreover, given also work challenges, rural breast cancer survivors may not take time off needed to travel for follow‐up cancer surveillance appointments 45. In one study, rural survivors were more likely to forgo medical and other care because of cost, especially older survivors 21. However, despite the lower level, OOP costs for rural survivors represent a substantial portion of income, especially for those more vulnerable.

The rural breast cancer survivors most burdened by cancer costs were younger and low‐income survivors. These results are in line with others who found that these groups, as well as women of minority backgrounds, are more likely to report worse financial‐related outcomes. For example, they are more likely to report a financial decline since diagnosis 38, debt and major privations such as less medical care or medication, no insurance, or utilities turned off because of missed bill payments 38, OOP cost burdens for medical care of 20% of income or higher 2, higher likelihood of financial hardship 36, 46, financial problems related to cancer 12, or higher economic hardship in survivorship such as difficulty living on household income 47. Therefore, understanding how support interventions may help containing the cancer cost burden is especially important for these most vulnerable groups.

Currently, to our knowledge, there are no effective interventions to address cost burden in cancer survivors. A recent systematic review summarized the rapidly evolving field of financial toxicity directly related to the continued and escalating costs of cancer care 48. The authors identified 45 relevant articles among 676 recent studies: Of these, none contained specific interventions to reduce the problem. However, the authors developed a multidimensional construct of financial hardship based on a typology of three related areas: material conditions (i.e., OOP costs, missed work days, reduced or lost income, and medical debt and bankruptcy); psychological response (i.e., distress related to cancer care costs and concern about wages and income to meet additional expenses); and coping behaviors (i.e., skipping or reducing medications, missing or delaying follow‐up cancer surveillance visits). Given this typology, future interventions may be directed toward understanding the material conditions, developing specific psychological response interventions, and evaluating the related coping behaviors. Data similar to those in our study are important to inform on material conditions related to patient/survivor‐reported cancer OOP costs for the period after primary treatment, and to identify the survivors most in need of intervention. Of interest is not only the OOP cost amounts, but what rural breast cancer survivors spent on: while medical care costs remained an important share of OOP costs for most survivors, one in five women spent increasing amounts on Counseling and Health Maintenance over time, and another one in about six women spent considerable OOP costs on Home Maintenance for help with everyday chores. With continued cost shifting to survivors through higher insurance premiums and deductibles, and the long‐term effects of cancer that require expenses in addition to medical care costs, strong assessment and ongoing surveillance of OOP costs and the multiple domains of hardship are warranted to ensure that survivors have access to the resources they need to maintain their health and well‐being.

There are some limitations to consider. First, OOP costs were self‐reported and were not systematically verified. However, the data collectors encouraged participants to have receipts and bills on hand to jog their memory about expenses incurred. Second, we may have not included some costs that survivors considered related to cancer. For example, a group of survivors reported expenses for gifts to people who helped them as costs they would not have had if they were not diagnosed with cancer 49, 50. Furthermore, we did not include costs associated with missing work due to pursuing medical care and other services to maintain health, as survivors may lose hourly wages. Third, we did not include a control group of participants without cancer; thus, we are not certain that the reported expenses are cancer related even though we asked participants to report breast cancer‐related expenses. However, other studies have shown higher OOP costs for patients with cancer compared to people with other chronic conditions 2. Furthermore, we are not certain about the costs that may have resulted from the interventions delivered in this study which educated women about the need for follow‐up care and the management of long‐term effects of cancer. Our participants may have incurred higher expenses than survivors not exposed to such education. Fourth, our participants may not represent rural cancer survivors in the United States or in Florida. However, by using a population‐based sampling we were able to recruit rural women living in all known rural counties in the State of Florida as well as those living in rural pockets of urban counties. All 432 participants indicated residence in one of 63 of the 67 counties in Florida.

## Conclusion

In this population of rural breast cancer survivors, the out‐of‐pocket costs of cancer‐related care persisted in the years post‐treatment and burdened especially younger and low‐income survivors. Research should continue to investigate the impact that costs of cancer may have on the ability of rural cancer survivors to afford follow‐up care and other services needed to optimize their well‐being, and develop interventions with a potential to address, at least in part, the long‐term economic burden of cancer.

## Conflict of Interest

The authors report no conflicts of interest.

## References

[1] Short, P. F. , J. R. Moran , and R. Punekar . 2011 Medical expenditures of adult cancer survivors aged <65 years in the United States. Cancer 117:2791–2800. 21656757 10.1002/cncr.25835 PMC4124459

[2] Bernard, D. S. , S. L. Farr , and Z. Fang . 2011 National estimates of out‐of‐pocket health care expenditure burdens among nonelderly adults with cancer: 2001 to 2008. J. Clin. Oncol. 29:2821–2826. 21632508 10.1200/JCO.2010.33.0522 PMC3139395

[3] Guy, G. P. Jr , D. U. Ekwueme , K. R. Yabroff , E. C. Dowling , C. Li , J. L. Rodriguez , et al. 2013 Economic burden of cancer survivorship among adults in the United States. J. Clin. Oncol. 31:3749–3757. 24043731 10.1200/JCO.2013.49.1241 PMC3795887

[4] Guy, G. P. Jr , K. R. Yabroff , D. U. Ekwueme , K. S. Virgo , X. Han , M. P. Banegas , et al. 2015 Healthcare expenditure burden among non‐elderly cancer survivors, 2008‐2012. Am. J. Prev. Med. 49:S489–S497. 26590644 10.1016/j.amepre.2015.09.002 PMC6051701

[5] Zheng, Z. , K. R. Yabroff , G. P. Guy , Jr., X. Han , C. Li , M. P. Banegas , et al. 2016 Annual medical expenditure and productivity loss among colorectal, female breast, and prostate cancer survivors in the United States. J. Natl Cancer Inst. 108: djw382. 10.1093/jnci/djv382 PMC4849808 26705361

[6] Davidoff, A. J. , M. Erten , T. Shaffer , J. S. Shoemaker , I. H. Zuckerman , N. Pandya , et al. 2013 Out‐of‐pocket health care expenditure burden for Medicare beneficiaries with cancer. Cancer 119:1257–1265. 23225522 10.1002/cncr.27848

[7] Pisu, M. , A. Azuero , K. Meneses , J. Burkhardt , and P. McNees . 2011 Out of pocket cost comparison between Caucasian and minority breast cancer survivors in the Breast Cancer Education Intervention (BCEI). Breast Cancer Res. Treat. 127:521–529. 20976542 10.1007/s10549-010-1225-0 PMC3087852

[8] Meneses, K. , A. Azuero , L. Hassey , P. McNees , and M. Pisu . 2012 Does economic burden influence quality of life in breast cancer survivors? Gynecol. Oncol. 124:437–443. 22138013 10.1016/j.ygyno.2011.11.038 PMC3278545

[9] Fenn, K. M. , S. B. Evans , R. McCorkle , M. P. DiGiovanna , L. Pusztai , T. Sanft . 2014 Impact of financial burden of cancer on survivors’ quality of life. J. Oncol. Pract. 10:332–338. 24865220 10.1200/JOP.2013.001322

[10] Kale, H. P. , and N. V. Carroll . 2016 Self‐reported financial burden of cancer care and its effect on physical and mental health‐related quality of life among US cancer survivors. Cancer 122:283–289. 26991528 10.1002/cncr.29808

[11] Ramsey, S. D. , A. Bansal , C. R. Fedorenko , D. K. Blough , K. A. Overstreet , V. Shankaran , et al. 2016 Financial insolvency as a risk factor for early mortality among patients with cancer. J. Clin. Oncol. 34:980–986. 26811521 10.1200/JCO.2015.64.6620 PMC4933128

[12] Kent, E. E. , L. P. Forsythe , K. R. Yabroff , K. E. Weaver , J. S. de Moor , J. L. Rodriguez . 2013 Are survivors who report cancer‐related financial problems more likely to forgo or delay medical care? Cancer 119:3710–3717. 23907958 10.1002/cncr.28262 PMC4552354

[13] McCullagh, P. , and J. Nelder . 1999 Generalized Linear Models, 2nd ed. Chapman & Hall/CRC Press, Boca Raton, FL.

[14] Weaver, K. E. , A. M. Geiger , L. Lu , and L. D. Case . 2013 Rural‐urban disparities in health status among US cancer survivors. Cancer 119:1050–1057. 23096263 10.1002/cncr.27840 PMC3679645

[15] Schootman, M. , S. Homan , K. E. Weaver , D. B. Jeffe , and S. Yun . 2013 The health and welfare of rural and urban cancer survivors in Missouri. Prev. Chronic. Dis. 10:E152. 24028832 10.5888/pcd10.130052 PMC3775393

[16] Andrykowski, M. A. , R. F. Steffens , H. M. Bush , and T. C. Tucker . 2014 Disparities in mental health outcomes among lung cancer survivors associated with ruralness of residence. Psychooncology 23:428–436. 24217966 10.1002/pon.3440

[17] Charlton, M. , J. Schlichting , C. Chioreso , M. Ward , and P. Vikas . 2015 Challenges of rural cancer care in the United States. Oncology (Williston Park). 29:633–640. 26384798

[18] Shugarman, L. R. , M. E. Sorbero , H. Tian , A. K. Jain , and J. S. Ashwood . 2008 An exploration of urban and rural differences in lung cancer survival among medicare beneficiaries. Am. J. Public Health 98:1280–1287. 17971555 10.2105/AJPH.2006.099416 PMC2424098

[19] Probst, J. C. , S. B. Laditka , J. Y. Wang , and A. O. Johnson . 2007 Effects of residence and race on burden of travel for care: cross sectional analysis of the 2001 US National Household Travel Survey. BMC Health Serv. Res. 7:40. 17349050 10.1186/1472-6963-7-40 PMC1851736

[20] Meilleur, A. , S. V. Subramanian , J. J. Plascak , J. L. Fisher , E. D. Paskett , and E. B. Lamont . 2013 Rural residence and cancer outcomes in the United States: issues and challenges. Cancer Epidemiol. Biomarkers Prev. 22:1657–1667. 24097195 10.1158/1055-9965.EPI-13-0404 PMC3814162

[21] Palmer, N. R. , A. M. Geiger , L. Lu , L. D. Case , and K. E. Weaver . 2013 Impact of rural residence on forgoing healthcare after cancer because of cost. Cancer Epidemiol. Biomarkers Prev. 22:1668–1676. 24097196 10.1158/1055-9965.EPI-13-0421 PMC3833446

[22] Katz, M. L. , P. L. Reiter , S. Corbin , J. S. de Moor , E. D. Paskett , and C. L. Shapiro . 2010 Are rural Ohio Appalachia cancer survivors needs different than urban cancer survivors? J. Cancer Surviv. 4:140–148. 20099044 10.1007/s11764-010-0115-0 PMC3650903

[23] Mathews, M. , R. West , and S. Buehler . 2009 How important are out‐of‐pocket costs to rural patients’ cancer care decisions? Can. J. Rural Med. 14:54–60. 19379628

[24] Meneses, K. , A. Azuero , X. Su , R. Benz , and P. McNees . 2014 Predictors of attrition among rural breast cancer survivors. Res. Nurs. Health 37:21–31. 24338864 10.1002/nur.21576 PMC4116629

[25] Meneses, K. M. , R. L. Benz , L. A. Hassey , Z. Q. Yang , and M. P. McNees . 2013 Strategies to retain rural breast cancer survivors in longitudinal research. Appl. Nurs. Res. 26:257–262. 24035222 10.1016/j.apnr.2013.08.001 PMC3836865

[26] Pisu, M. , K. Meneses , A. Azuero , R. Benz , X. Su , and P. McNees . 2016 Variation in resources needed to implement psychosocial support interventions for rural breast cancer survivors. J. Cancer Surviv. 10:375–383. 26341349 10.1007/s11764-015-0483-6 PMC4779072

[27] McNees, P. , and K. Meneses . 2012 Index of research access. Nursing Research and Reviews. 3:5–7.

[28] Given, B. A. , C. W. Given , and M. Stommel . 1994 Family and out‐of‐pocket costs for women with breast cancer. Cancer Pract. 2:187–193. 8055022

[29] Meneses, K. D. , P. McNees , V. W. Loerzel , X. Su , Y. Zhang , and L. A. Hassey . 2007 Transition from treatment to survivorship: effects of a psychoeducational intervention on quality of life in breast cancer survivors. Oncol. Nurs. Forum 34:1007–1016. 17878129 10.1188/07.ONF.1007-1016

[30] Hann, D. , K. Winter , and P. Jacobsen . 1999 Measurement of depressive symptoms in cancer patients: evaluation of the Center for epidemiological studies depression scale (CES‐D). J. Psychosom. Res. 46:437–443. 10404478 10.1016/s0022-3999(99)00004-5

[31] Radloff, L. 1977 The CES‐D scale: a self‐report depression scale for research in the general population. Appl. Psychol. Meas. 1:385–401.

[32] Azuero, A. , M. Pisu , P. McNees , J. Burkhardt , R. Benz , and K. Meneses . 2010 An application of longitudinal analysis with skewed outcomes. Nurs. Res. 59:301–307. 20585226 10.1097/NNR.0b013e3181e507f1 PMC2929564

[33] Nekhlyudov, L. , R. Walker , R. Ziebell , B. Rabin , S. Nutt , and J. Chubak . 2016 Cancer survivors’ experiences with insurance, finances, and employment: results from a multisite study. J. Cancer Surviv. 10:1104–1111. 27277896 10.1007/s11764-016-0554-3

[34] Banegas, M. P. , G. P. Jr Guy , J. S. de Moor , D. U. Ekwueme , K. S. Virgo , E. E. Kent , et al. 2016 For working‐age cancer survivors, medical debt and bankruptcy create financial hardships. Health Aff. (Millwood). 35:54–61. 26733701 10.1377/hlthaff.2015.0830 PMC6057727

[35] Whitney, R. L. , J. F. Bell , S. C. Reed , R. Lash , R. J. Bold , K. K. Kim , et al. 2016 Predictors of financial difficulties and work modifications among cancer survivors in the United States. J. Cancer Surviv. 10:241–250. 26188363 10.1007/s11764-015-0470-y PMC4854648

[36] Yabroff, K. R. , E. C. Dowling , G. P. Jr Guy , M. P. Banegas , A. Davidoff , X. Han , et al. 2016 Financial hardship associated with cancer in the United States: findings from a population‐based sample of adult cancer survivors. J. Clin. Oncol. 34:259–267. 26644532 10.1200/JCO.2015.62.0468 PMC4872019

[37] Zajacova, A. , J. B. Dowd , R. F. Schoeni , and R. B. Wallace . 2015 Employment and income losses among cancer survivors: estimates from a national longitudinal survey of American families. Cancer 121:4425–4432. 26501494 10.1002/cncr.29510 PMC4670608

[38] Jagsi, R. , J. A. Pottow , K. A. Griffith , C. Bradley , A. S. Hamilton , J. Graff , et al. 2014 Long‐term financial burden of breast cancer: experiences of a diverse cohort of survivors identified through population‐based registries. J. Clin. Oncol. 32:1269–1276. 24663041 10.1200/JCO.2013.53.0956 PMC3986387

[39] Ubel, P. , A. P. Abernethy , and Y. S. Zafar . 2013 Full disclosure ‐ out‐of‐pocket costs as side effects. N. Engl. J. Med. 369:1484–1486. 24131175 10.1056/NEJMp1306826

[40] Lewis Dolan, P . 2011 Oncologists confront “financial toxicity” of cancer care. American Medical News (amednews.com) http://pnhp.org/blog/2011/06/20/financial-toxicity-of-cancer-and-ahrqs-effective-health-care-program/.

[41] Tucker‐Seeley, R. D. , and K. R. Yabroff . 2016 Minimizing the “financial toxicity” associated with cancer care: advancing the research agenda. J. Natl Cancer Inst. 108:djv410. 10.1093/jnci/djv410 26657336

[42] Zafar, S. Y. , and A. P. Abernethy . 2013 Financial toxicity, Part I: a new name for a growing problem. Oncology (Williston Park). 27:149. PMC4523887 23530397

[43] Zafar, S. Y .2016 Financial Toxicity of Cancer Care: It's Time to Intervene. J. Natl Cancer Inst. 108:djv370. 10.1093/jnci/djv370 26657334

[44] Institute of Medicine . 2013 Delivering high‐quality cancer care: charting a new course for a system in crisis. The National Academies Press, Washington, DC. 24872984

[45] Gray, R. E. , M. Fitch , M. Greenberg , A. Hampson , M. Doherty , and M. Labrecque . 1998 The information needs of well, longer‐term survivors of breast cancer. Patient Educ. Couns. 33:245–255. 9731162 10.1016/s0738-3991(98)00024-x

[46] Shankaran, V. , S. Jolly , D. Blough , and S. D. Ramsey . 2012 Risk factors for financial hardship in patients receiving adjuvant chemotherapy for colon cancer: a population‐based exploratory analysis. J. Clin. Oncol. 30:1608–1614. 22412136 10.1200/JCO.2011.37.9511

[47] Pisu, M. , K. M. Kenzik , R. A. Oster , P. Drentea , K. T. Ashing , M. Fouad , et al. 2015 Economic hardship of minority and non‐minority cancer survivors 1 year after diagnosis: another long‐term effect of cancer? Cancer 121:1257–1264. 25564986 10.1002/cncr.29206 PMC4393356

[48] Altice, C. K. , M. P. Banegas , R. D. Tucker‐Seeley , and K. R. Yabroff . 2017 Financial hardships experienced by cancer survivors: a systematic review. J. Natl Cancer Inst. 109:djw205. 10.1093/jnci/djw205 PMC6075571 27754926

[49] Moore, K. 1998 Out‐of‐pocket expenditures of outpatients receiving chemotherapy. Oncol. Nurs. Forum 25:1615–1622. 9802056

[50] Moore, K. A. 1999 Breast cancer patients’ out‐of‐pocket expenses. Cancer Nurs. 22:389–396. 10526432 10.1097/00002820-199910000-00007

